# Study Protocol on Intentional Distortion in Personality Assessment: Relationship with Test Format, Culture, and Cognitive Ability

**DOI:** 10.3389/fpsyg.2016.00933

**Published:** 2016-06-28

**Authors:** Eline Van Geert, Altan Orhon, Iulia A. Cioca, Rui Mamede, Slobodan Golušin, Barbora Hubená, Daniel Morillo

**Affiliations:** ^1^Faculty of Psychology and Educational Sciences, KU Leuven (University of Leuven)Leuven, Belgium; ^2^Department of Psychology, Istanbul Bilgi UniversityIstanbul, Turkey; ^3^ScienceForWorkMilan, Italy; ^4^Formerly affiliated with Faculty of Psychology and Education Sciences, University of CoimbraCoimbra, Portugal; ^5^Faculty of Philosophy, University of Novi SadNovi Sad, Serbia; ^6^Department of Psychology, Faculty of Arts, Masaryk UniversityBrno, Czech Republic; ^7^Chair of Psychometric Models and Applications, Department of Social Psychology and Methodology, Faculty of Psychology, Autonomous University of MadridMadrid, Spain

**Keywords:** personality assessment, personnel selection, forced-choice, Thurstonian IRT, faking, ipsativity, cross-cultural comparison

## Abstract

Self-report personality questionnaires, traditionally offered in a graded-scale format, are widely used in high-stakes contexts such as job selection. However, job applicants may intentionally distort their answers when filling in these questionnaires, undermining the validity of the test results. Forced-choice questionnaires are allegedly more resistant to intentional distortion compared to graded-scale questionnaires, but they generate ipsative data. Ipsativity violates the assumptions of classical test theory, distorting the reliability and construct validity of the scales, and producing interdependencies among the scores. This limitation is overcome in the current study by using the recently developed Thurstonian item response theory model. As online testing in job selection contexts is increasing, the focus will be on the impact of intentional distortion on personality questionnaire data collected online. The present study intends to examine the effect of three different variables on intentional distortion: (a) test format (graded-scale versus forced-choice); (b) culture, as data will be collected in three countries differing in their attitudes toward intentional distortion (the United Kingdom, Serbia, and Turkey); and (c) cognitive ability, as a possible predictor of the ability to choose the more desirable responses. Furthermore, we aim to integrate the findings using a comprehensive model of intentional distortion. In the Anticipated Results section, three main aspects are considered: (a) the limitations of the manipulation, theoretical approach, and analyses employed; (b) practical implications for job selection and for personality assessment in a broader sense; and (c) suggestions for further research.

## Introduction

Self-report personality questionnaires are increasingly popular in high-stakes contexts such as personnel selection (Rothstein and Goffin, 2006), college admissions (Sjöberg, 2015), and determining eligibility to stand trial (Archer et al., 2006). However, in these situations, instead of answering honestly, test takers often intentionally distort their answers to increase their chances of being hired (Birkeland et al., 2006). It has been estimated that roughly 30 percent of job applicants intentionally distorts their responses (Griffith and Converse, 2011). Intentional distortion is detrimental to the psychometric properties of the assessment instrument, hiring decisions, and the utility of whole-job selection systems (Donovan et al., 2014), although human resources practitioners are largely unaware of the implications (Rothstein and Goffin, 2006). Furthermore, research on intentional distortion suffers from weak theoretical support and over-reliance on empirical and statistical methods (Griffith and Peterson, 2011).

In the literature there is considerable debate on the question whether intentional distortion also decreases the predictive validity of self-report questionnaires. Donovan et al. (2014) conducted a study utilizing a within-subjects design in an actual organizational setting and found not only a negative impact of intentional distortion on the psychometric properties of the non-cognitive self-report measure, but also a negative impact on the quality of the hiring decisions made by the organization. Additionally, people intentionally distorting their answers were found to exhibit lower levels of performance than people answering honestly. This implies that intentional distortion has negative consequences for the predictive validity of the personality test. The opposite argument, however, is based on seeing intentional distortion as a type of intelligence, mostly related to social or emotional intelligence, which consists of the ability to correctly read and interpret cues in social situations. This ability allows test takers to identify correctly the meaning of the test items and the desirable characteristics for the job in question, and later on will also help them perform better at their job, especially if it involves social interactions (Kleinmann et al., 2011). Thus, in this view, the influence of intentional distortion on the personality test leads to an equal or increased predictive validity of the test.

Even when intentional distortion would lead to a better predictive or criterion-related validity of personality tests, it is also important to consider the construct validity of the test. If the test does not measure what it is expected to measure, in this case personality factors, then the construct validity is low. Understanding and reducing the influence of intentional distortion on these measures of personality should lead toward an ideal situation in which a personality test assesses personality (and not intentional distortion), and another test assesses intentional distortion or a related ability, if this variable would have predictive validity for job performance (Kleinmann et al., 2011).

**FIGURE 1:**
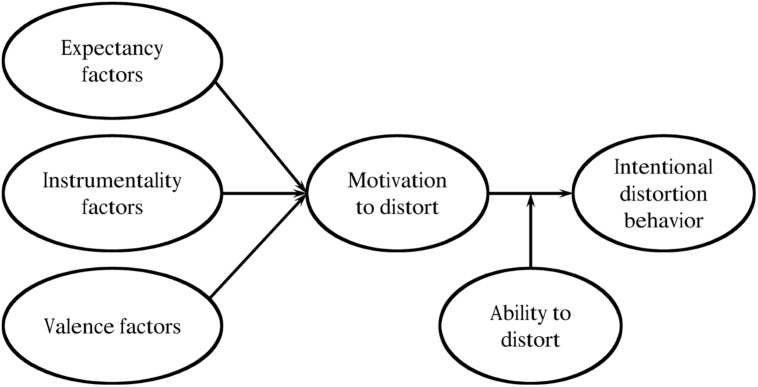
**Graphical representation of the valence-instrumentality-expectancy theory, contextualized for intentional distortion behavior.** The proximal determinants of the motivation to distort and intentional distortion behavior moderated by the ability to distort are represented. In Ellingson and McFarland (2011). Copyright 2011 by the Taylor & Francis Group, LLC. Reprinted with permission.

The most comprehensive theoretical model of intentional distortion (see **Figure 1**; Ellingson and McFarland, 2011) is based on the valence-instrumentality-expectancy theory of motivation (Vroom, 1964). This model states that the predictors of intentional distortion work through three proximal motivational factors: (a) valence, the affective reaction an individual has to a particular outcome of an action; (b) instrumentality, the belief that the action will lead to a particular outcome; and (c) expectancy, the belief that one can perform the action. These three factors determine a person’s motivation to engage in intentional distortion; however, the individual’s actual ability to enact intentional distortion moderates the effect of the motivation to do so (Ellingson and McFarland, 2011).

Situational characteristics such as test format may offset individuals’ capacities for intentional distortion. Forced-choice response formats have been proposed as a way of controlling for intentional distortion in personality assessments (Christiansen et al., 2005). In forced-choice questionnaires, instead of rating items on a graded scale, respondents rank groups of personality statements that seem equivalent in terms of social desirability. Forced-choice questionnaires hinder the identification of advantageous response patterns (Stark et al., 2014), rule out uniform biases such as acquiescence and extreme responding, and are recommended for use in cross-cultural comparisons and high-stakes situations (He et al., 2014). On the other hand, another type of scale format, that of dichotomous answers (yes/no) is rarely used (e.g., Eysenck Personality Questionnaire, Eysenck and Eysenck, 1975), being advantageous in terms of time, it takes to complete the test. However, problems arise with extremely unbalanced response distributions (e.g., everyone answers “yes” to a certain item; Clark and Watson, 1995) which indicates intentional distortion, and the measurement of continuous personality variables through completely polarized items, which minimizes the information obtained for those with real scores in extremities of the distribution (Furr, 2011).

Despite their advantages, forced-choice questionnaires have traditionally been discounted due to problems arising from conventional approaches to scoring them, which produce ipsative scores. These are able to show the relative levels of a trait within an individual (e.g., an individual appears more agreeable than conscientious), but they cannot be used to compare absolute levels between individuals (Christiansen et al., 2005). An increase on one dimension in an ipsative measurement necessitates a corresponding decrease on other dimensions. This property makes ipsative measures incompatible with methods such as correlation or factor analysis (Cornwell and Dunlap, 1994) and creates issues relating to construct validity, criterion-related validity, and reliability estimates (Brown and Maydeu-Olivares, 2013). Hicks (1970, p. 181) cautioned researchers against using purely ipsative instruments, writing, “[researchers] cannot legitimately manipulate the variables assessed by the test utilizing statistical procedures which assume that independent dimensions are involved.” Methods proposed to address this issue have included increasing the number of dimensions being measured (Hicks, 1970) and compositional data analysis (Aitchison and Egozcue, 2005), yet the relative nature of the inferences remained unresolved (van Eijnatten et al., 2015). However, recent models based on IRT allow the extraction of normative scores from forced-choice responses (Stark et al., 2014; Joubert et al., 2015). Among these, the two state-of-the-art models are the Thurstonian IRT model (Brown and Maydeu-Olivares, 2011) and multi-unidimensional pairwise preference model (MUPP; Stark et al., 2005). These models overcome the problems posed by scoring ipsative measures via classical test theory by explicitly proposing a measurement model, that describes the relationship between items and traits, and a decision model, that describes how the individual selects one item over another (Brown, 2016). This additional level of information is what allows the recovery of normative scores from a forced-choice instrument – on Thurstonian IRT, a structure of correlated latent factors derived from multiple blocks of forced-choice items is fitted to binary outcomes of pairwise comparisons (Brown and Maydeu-Olivares, 2013), whereas MUPP combines multidimensional items with unidimensional pairings and a Bayes modal procedure as means of estimating trait scores (Stark et al., 2005).

The aim of our study is twofold: (a) to present an integrated view of intentional distortion formulated on sound theoretical underpinnings and (b) to reduce the effects of intentional distortion on personality assessment in high-stakes contexts by testing a viable method of scoring forced-choice questionnaires that can overcome earlier difficulties in their use (i.e., the ipsativity problem). Along with this, we will investigate three variables previously found to affect intentional distortion and present the theoretical reasoning behind these hypothesized effects.

First, responses for forced-choice questionnaires should exhibit lower levels of intentional distortion than those for graded-scale questionnaires. Besides the effects of forced-choice format on the ability to distort discussed above (i.e., more difficult identification of advantageous response patterns), having to choose between statements with similar levels of social desirability could induce higher levels of test-taking anxiety in applicants (Converse et al., 2008), lowering cognitive performance and ability to distort. According to Converse et al. (2008), this may come from a perception that in forced-choice format they do not have free choice of answers as well as less opportunity to express their personality qualities related to the job. Additionally, the forced-choice format could diminish their expectancy beliefs about intentional distortion of their answers (Ellingson and McFarland, 2011).

Second, attitudes toward the appropriateness of a candidate’s use of intentional distortion are associated with several cultural dimensions suggested by the GLOBE study (House et al., 2004), such as uncertainty avoidance (which decreases the appropriateness due to lack of control about the result), or power distance (enhancing the appropriateness due to perceived lack of fairness in societies high in power distance; Fell et al., 2015). These attitudes may act on intentional distortion through (a) valence beliefs, by informing personal standards of behavior, or (b) instrumentality beliefs, by leading to the belief in a more positive outcome of intentional distortion (Ellingson and McFarland, 2011).

Third, because forced-choice questionnaires are more cognitively demanding compared to graded-scale questionnaires (Converse et al., 2008), intentional distortion is expected to relate more strongly to cognitive ability in forced-choice questionnaires than in graded-scale questionnaires. Cognitive ability is on one hand expected to relate positively to the ability of applicants to distort their answers (Christiansen et al., 2005), as it is assumed that more cognitively able applicants will be better able to identify advantageous response patterns in relation to the job requirements. On the other hand, there has also been evidence showing that people with higher cognitive ability distort their answers less often (Austin et al., 2002; Levashina et al., 2009) and do not respond in a more socially desirable manner than other participants (Ones et al., 1996). Reasons for avoiding intentional distortion of their answers include high self-efficacy and believing in one’s own abilities to succeed in assessment without distorting (De Fruyt et al., 2006), short-term outcomes (such as being excluded from the applicants pool for failing social desirability items), or long-term outcomes (such as not being suitable for the role or not fitting into the working team). However, if this would be the case, this relationship would also be evident in the graded-scale questionnaires.

Consequently, our research questions are as follows:

1. Is intentional distortion lower in forced-choice questionnaires than in graded-scale questionnaires?
2. Are there differences in levels of intentional distortion across cultural groups?
3. Do people with higher cognitive ability show more intentional distortion in forced-choice questionnaires than people with lower cognitive ability?

## Materials and Equipments

### Measures

#### Big Five Inventory

The BFI is a popular instrument for international studies and it is recommended for use in cross-cultural settings (Schmitt et al., 2007). This 44-item graded-scale-format operationalization (Pervin and John, 1999) of the Big Five theory (John et al., 2008) will be used to assess personality traits. Adaptations of the BFI to the languages of the target countries, as well as country-specific psychometric properties, are available (Schmitt et al., 2007; Neşe Alkan, “Reliability and Validity of the Turkish Version of the Big Five Inventory,” unpublished manuscript, 2006).

#### Tailored Forced-Choice Questionnaires

Equivalent forced-choice questionnaires for each country will be constructed by pairing positively keyed items measuring personality traits from the International Personality Item Pool (Goldberg, 1999). Each Big Five trait is represented by 30 items that were selected to reflect the diversity of their respective domains.

In order to ensure that the items being paired to form the blocks in the forced-choice questionnaire are as closely matched in social desirability as possible, we are conducting a study to gage social desirability levels for each item. Approximately 250 respondents (as in Stark et al., 2005) in each country will be asked to rate the items for their attractiveness. Given that social desirability may be a context-dependent property (Rothstein and Goffin, 2000), the participants will be presented with the job description of the high-stakes condition and prompted to rate the social desirability “as if” applying for that job. Next, the items will be paired using a procedure that (a) generates a list of possible pairs of items on different dimensions of the Big Five, numbering 10 pairs initially; (b) sorts the items from most desirable to least desirable, according to mean ratings; (c) identifies the two items whose means are closest; (d) removes the pair constituted by the two items from the search space; and (e) repeats the process of pairing the closest items until no more pairs remain in the search space, after which the procedure enters the next round of matching. Pairing 150 items in this manner requires eight rounds.

#### International Cognitive Ability Resource

We will use the 16-item ICAR Sample Test (The International Cognitive Ability Resource Team, 2014) to measure cognitive ability. This instrument, designed for online administration (Condon and Revelle, 2014), is a public-domain measure with four subscales: Letter-Number Series, Matrix Reasoning, 3D Rotation, and Verbal Reasoning. The test has been adapted for use in the native languages of the countries in this study. (Scores will be used for within-culture comparisons only.) Condon and Revelle (2014) report adequate internal consistency for the ICAR Sample Test (Cronbach’s α = 0.81, total ω = 0.83) and provide evidence of adequate convergent validity with several widely accepted measures of cognitive ability.

## Stepwise Procedures

### Participants

Data will be collected from university students or recent graduates in their early adulthood (aged 18–30) in three countries: the United Kingdom, Serbia, and Turkey. Approximately 250 participants from each country will take part in the study to construct the tailored forced-choice questionnaires and 500 participants from each country will take part in the experimental study. They will be recruited online (mostly resorting to social media, e.g., Facebook, Twitter), by using university resources (i.e., using online subject pool programs or by administering them to students during or after classes), and by advertising the study in university facilities. In order to maximize participation, the advertisements will be timed to avoid periods that would be associated with decreased participation. The participants of the experimental study will be motivated by the opportunity to enter a raﬄe for financial prizes and the opportunity to get individual feedback on their personality.

The targeted group of participants are students and graduates in their early adulthood according to Erikson’s (1993) stage of human development. This stage is, besides completing formation adult identity and establishing intimate relationships, typical of finishing one’s education and entering the job market. University students and fresh graduates are likely to be familiar with the situation of applying for jobs, going through job interviews and assessment, including personality assessment. Moreover, the role of assistant manager which is used to introduce the high-stakes condition might be quite realistic and relatively attractive job for wide range of university students and fresh graduates of different specializations with limited work experience (Kleinmann and Klehe, 2011).

Participating countries were chosen based on differences in attitudes toward intentional distortion in job interviews (Fell et al., 2015), which were related to the cultural dimensions (e.g., power distance, in-group collectivism) assessed by the international GLOBE study (House et al., 2004). Our choices are representative of presumed minimum, intermediate, and maximum levels on this attitude index (the United Kingdom, Serbia, and Turkey, respectively), on which a higher score represents a more positive attitude toward intentional distortion. Although Serbia was not included in the GLOBE study, later research provided information on the cultural dimensions in question (Vukonjanski et al., 2012).

### Ethics Statement

The study has been given full clearance by the Ethics Committee of Universidad Autónoma de Madrid, which abides law 14/2007 of July 3, 2007 regarding biomedical research, and is fully compliant with the Declaration of Helsinki.

### Design and Procedure

**FIGURE 2:**
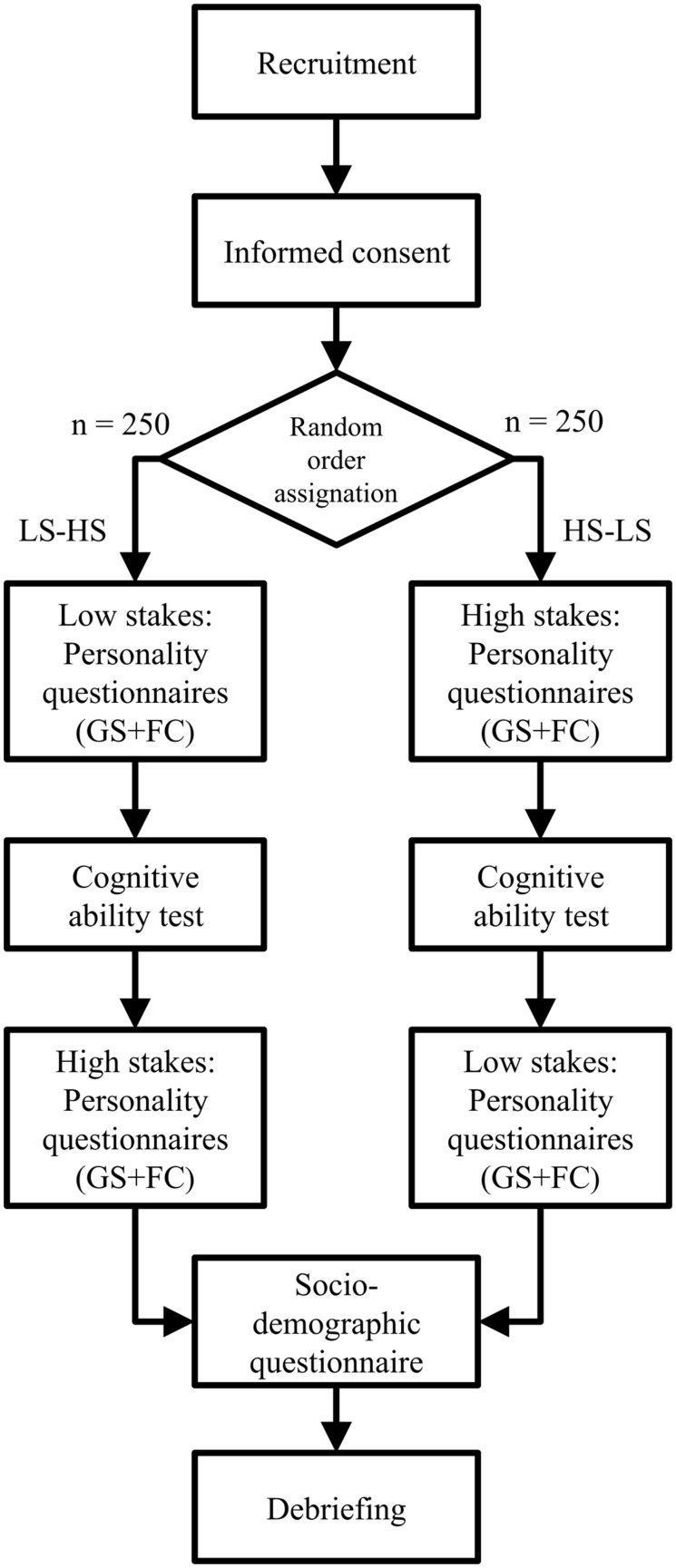
**Research design flowchart.** LS, low stakes; HS, high stakes; GS, graded scale; FC, forced choice.

Participants will be invited to take a set of online tests in a single session. The tests will be administered via the Qualtrics platform (Qualtrics, Provo, UT, USA). The set includes two self-report questionnaires (graded-scale and forced-choice format), each administered in two conditions (high-stakes and low-stakes), and a test of cognitive ability. In the low-stakes condition, participants will be instructed to respond as sincerely as possible. In the high-stakes condition, they will be instructed to answer as if they were applying for a job—in this case, a management trainee position. Every participant will go through both the high-stakes and the low-stakes condition, with order determined by random assignment (see **Figure 2**). The within-subject design is recommended for studying intentional distortion because it accounts for individual tendencies in the behavior (Viswesvaran and Ones, 1999). Between the two conditions, respondents will answer a cognitive ability measure, which should have the additional benefit of reducing practice or memory effects for the questionnaires (Grieve and de Groot, 2011). Finally, respondents will answer a single item asking to what extent the described job is attractive for individual participants on a four-point scale (from very unattractive to very attractive). This will allow us to operationalize job attractiveness and possibly control for it. In return for participation, respondents who complete the whole questionnaire will have the possibility to participate in a raﬄe containing several monetary reward. The participants will also be offered personalized feedback based on the BFI scores in the low-stakes condition which should increase the respondents’ motivation to answer the questionnaire in an accurate and honest manner under this instruction.

### Proposed Analysis

The Thurstonian IRT model (Brown and Maydeu-Olivares, 2011) has proved to be a flexible, robust model for obtaining normative trait scores from individual responses to forced-choice questionnaires. In contrast to the MUPP (Stark et al., 2005), it does not require precalibration of the item parameters. It can be estimated using the widespread software Mplus (Muthén and Muthén, 2015) and thus does not require any specialized software. Finally, the International Personality Item Pool items that will be used in the forced-choice questionnaires are written in a way that assumes a dominance response model, in which an individual is more likely to answer positively to items assessing traits they are high on; as such, these items are better fit by the Thurstonian IRT than an unfolding model such as the MUPP (Brown and Maydeu-Olivares, 2010). Therefore, Thurstonian IRT is the model of choice to analyze the ipsative data.

This model is based on Thurstone’s (1927) Law of Comparative Judgement. It links the utility of each response option to the latent trait it intends to measure, by means of a linear function (Brown and Maydeu-Olivares, 2011). As a result, the probability that a respondent chooses item *i* in a binary comparison between items *i* and *k* in block *l* is expressed by (p. 473),

$$\text{P}\left(Y_{l}=1|\eta_{a},\;\eta_{b}\right)=\text{Φ}\left(\frac{-\gamma_{l}+\lambda_{i}\eta_{a}+\lambda_{k}\eta_{b}}{\sqrt{\psi_{i}^{2}+\psi_{k}^{2}}}\right)\text{,}$$

where Φ_(*x*)_ is the cumulative standard normal distribution function at *x*, γ_l_ is the threshold for the binary comparison of the two items block *l*, λ_*i*_ and λ_*k*_ are the factor loadings of items *i* and *k* on their respective factors *a* and *b*, $\psi_{i}^{2}$ and $\psi_{k}^{2}$ the unique variances of items *i* and *k*, and η_a_ and η_b_ a respondent’s scores in factors *a* and *b*, respectively. By fitting a confirmatory factor-analytic model to the data, item and population parameters can be estimated for the model. Then, normative person parameters can be obtained through a maximum *a posteriori* estimator. Brown and Maydeu-Olivares (2012) provide and document an Excel macro that can be used to generate the necessary input files to Mplus for a given forced-choice questionnaire – the output allows estimation and scoring according to a Thurstonian IRT model that fits the data, computing item loadings, item thresholds, and factor scores.

The Thurstonian IRT model will be integrated into a wider structural equation model, where the responses to the forced-choice questionnaire and the graded-scale questionnaire will be jointly modeled. The bivariate information from the low-stakes condition will then be used to fit the structural equation model, and an invariance analysis will be conducted to check for invariance of the two order conditions. Then, a multitrait-multimethod matrix will be assessed for construct, convergent, and discriminant validity. After that, *maximum a posteriori* scores for the respondents’ latent traits in both conditions will be obtained using Mplus (Brown and Maydeu-Olivares, 2012).

Two intentional distortion scores will be obtained for each respondent by subtracting the IRT-based trait scores in the low-stakes (reference score) from those in the high-stakes condition: one concerning each test format (graded-scale versus forced-choice). To answer the first research question, those intentional distortion scores will be tested for significant differences using Rao’s *F*-test (Christiansen et al., 2005). To test the second research question, intentional distortion scores of the three cultural samples will be tested for differences across country groups using an analysis of variance test (Converse et al., 2010). Finally, a linear regression analysis will be conducted between intentional distortion scores and cognitive ability scores to answer the third research question.

## Anticipated Results

The present study intends to clarify the influence of test format, culture, and cognitive ability on intentional distortion in self-report personality measures. Hypotheses made concerning the influence of test format, culture, and cognitive ability are based on and integrated in the theoretical model of intentional distortion by Ellingson and McFarland (2011). However, the proposed underlying processes are still to be tested in further research.

Firstly, tests that use a forced-choice item format have been proposed to reduce the effects of respondents’ intentional distortion on the test results. However, they have proved to be impractical up to now because forced-choice questionnaire items generate ipsative data. By using an IRT-based data analysis, the present study aims to increase the applicability of the forced-choice tests, and provide a valuable alternative for practitioners to reduce the effects of intentional distortion in personality assessment. As forced-choice format makes it more difficult to identify advantageous response patterns (Stark et al., 2014) and might also decrease expectancy beliefs (i.e., belief in ability to successfully distort), it is expected that intentional distortion will be lower in forced-choice questionnaires than in graded-scale questionnaires.

The results of our study regarding the test format will be of practical relevance for the assessment in high-stakes situations, such as personnel selection, where important decisions are made based on candidates’ scores on personality tests. Future research could explore the utility of the assessment method for other high-stakes contexts, such as establishing eligibility for trial. In the long term, this will enable a more accurate and fairer assessment of participants in high-stakes contexts.

Secondly, it is expected that cultures differ in the extent of intentional distortion they display. More specifically, it is expected that participants from cultures scoring low, medium, or high on the index of positive attitude toward intentional distortion (the United Kingdom, Serbia, and Turkey, respectively), will show, respectively, low, medium, and high levels of intentional distortion. This influence of culture on intentional distortion may act through valence beliefs (i.e., informing personal attitude toward intentional distortion) and instrumentality beliefs (i.e., affecting belief that intentional distortion will lead to positive outcomes; Ellingson and McFarland, 2011).

Cross-national work-related mobility is increasing nowadays, and likewise with the reach of multinational enterprises. Practitioners conducting personality assessment in such cross-national contexts need to understand the differences in their respondents’ tendencies to complete personality tests in certain ways. By investigating the phenomenon of intentional distortion in three countries that differ in their attitude toward this practice, the present study will have further implications for international assessment.

Thirdly, we will also explore the relationship between a person’s general cognitive ability and intentional distortion, both on graded-scale and forced-choice items. In graded-scale questionnaires, no influence of cognitive ability on intentional distortion is expected. In forced-choice questionnaires, a positive relation of cognitive ability and the ability to distort is hypothesized, as it is expected that participants should be more able to identify the advantageous response patterns. Moreover, cognitive ability might also reinforce a person’s motivation to distort by raising their expectancy beliefs of how successful they will be at distorting their answers.

Nevertheless, a potential rejection of this hypothesis could indicate support for an alternative explanation. Participant’s cognitive ability can be negatively related to their motivation to distort as more cognitively able applicants would be more aware of possible short-term consequences (such as being excluded from the applicants pool for failing social desirability items), or long-term consequences (such as not being suitable for the role or not fitting into working team) of distorting answers in high-stakes contexts. Yet another reason for choosing not to distort in participants with high cognitive skills is associated with higher self-efficacy and belief that they can score high without distorting (Levashina et al., 2009), so their expectancy belief may be that distorting is not worth the effort and risk-taking. However, because of the simulated nature of the high-stakes manipulation, the motivational processes to distort may differ from those in an actual high-stakes situation, for example because the long-term consequences are less taken into consideration, which threatens the ecological validity of the results. Simulating the high-stakes situations is a common practice in this field of research (see, e.g., Christiansen et al., 2005), but future studies with real job applicants would be recommended to validate our findings and their applicability in real-life situations. Additionally, although the nature of the specific instruction set given in the high-stakes context (i.e., “respond as if applying for a job”) was chosen to be as ecologically valid as possible in a simulated context, this instruction set does not distinguish between the short- and long-term consequences possibly influencing the motivation to distort, thereby compromising internal and external validity. To disentangle both motivations, further studies could include an additional high-stakes condition focusing on short-term consequences specifically (e.g., “respond so as to maximize your chances of getting hired”).

Understanding how cognitive ability and intentional distortion relate in the context of assessment is important to clarify aspects of predictive and construct validity of personality tests. Although a high predictive validity is useful in practice, it is essential to understand what the test actually measures. We have tried to achieve this by anchoring the study design in a solid theoretical framework that not only contributes to explaining the interrelations between concepts but also can guide future research to build a deeper and more comprehensive understanding of intentional distortion.

Limitations of our experimental design include the use of student groups as representative populations, lack of control over the physical testing environment and sample equivalence, and the possibility of a high rate of attrition leading to less diversity in sample. However, we try to mitigate the effect of the first aspect by advertising the study to recent graduates and students in final years, who are confronting (or will soon be confronting) the challenge of obtaining their first job. Regarding control over the physical environment, online assessment is an increasingly common practice, with 81% of the companies that use assessment administering it online (Kantrowitz, 2014), despite its potential disadvantages. Furthermore, it appears that online tests and pen-and-paper versions are roughly equal in their susceptibility to intentional distortion (Grieve and de Groot, 2011); therefore, research on intentional distortion in online assessment is still needed. Weigold et al. (2013) describe two studies comparing results for surveys administered via traditional means (e.g., on paper and in lab settings) and surveys administered either online or in a take-home format. The instruments used in these studies purportedly measured personality and social desirability. The authors reported that paper-and-pencil and online survey administration were generally equivalent except for some auxiliary aspects such as response rates and completion time. However, Joinson (1999) described an effect whereby participants reported lower social anxiety and social desirability influence in an online survey compared to a paper-based survey, and when they were anonymous compared to being identified. In the case of the present study, it is expected that most participants will provide some personally identifying information in the course of enrolling for the raﬄe. The present study attempts to reproduce the conditions of high-stakes assessment in a job selection context. Having participants identify themselves matches more closely the conditions of real-life job selection, and a hypothetical increase in susceptibility to social desirability likewise matches what, we intend to study. Because of this, our choice of methodology might be more appropriate for drawing conclusions for this type of assessment.

## Author Contributions

All authors listed, have made substantial, direct and intellectual contribution to the work, and approved it for publication.

## Conflict of Interest Statement

The authors declare that the research was conducted in the absence of any commercial or financial relationships that could be construed as a potential conflict of interest.

## Abbreviations

BFI: big five inventory
ICAR: international cognitive ability resource
IRT: item-response theory
MUPP: multi-unidimensional pairwise preference [model]
